# The Contribution of Multiple Pregnancies in Stillbirths in Greece: A Time-Trend Analysis

**DOI:** 10.7759/cureus.54628

**Published:** 2024-02-21

**Authors:** Nikolaos Vlachadis, Sofoklis Stavros, Nikolaos Machairiotis, Dionysios Vrachnis, Nikolaos Loukas, Nikolaos Antonakopoulos, Alexandros Fotiou, Georgios Maroudias, Petros Drakakis, Nikolaos Vrachnis

**Affiliations:** 1 Department of Obstetrics and Gynecology, General Hospital of Messinia, Kalamata, GRC; 2 Third Department of Obstetrics and Gynecology, National and Kapodistrian University of Athens, Attiko Hospital, Athens, GRC; 3 Department of Obstetrics and Gynecology, University of Patras, Rio Hospital, Patras, GRC

**Keywords:** perinatal mortality, multiple births, greece, trend analysis, stillbirth

## Abstract

Introduction

Multiple pregnancy is an established risk factor for fetal death. This study aimed to examine the impact of multifetal pregnancies on stillbirth rates (SBRs) in the Greek population.

Methods

Data on live births and stillbirths by multiplicity were derived from the Hellenic Statistical Authority, covering a 65-year period from 1957 to 2021. The SBR for multiple and single gestations, and the population attributable risk (%) (PAR (%)) stillbirth attributable to multifetal gestations were calculated, and temporal trends were assessed using joinpoint regression analysis, with annual percentage changes (APC) and 95% confidence interval (95% CI).

Results

In the period 1957-2021, multiple pregnancies accounted for 9.4% of total stillbirths in Greece and the overall relative risk of fetal death among multifetal gestations was 3.34, in comparison with singletons. The SBR in multiple births remained unchanged from 1957 to 1976 and showed downward trends from 1976 to 2021 (APC = -3.0, 95% CI: -3.4 to -2.7, p < 0.001). PAR (%), after two decades of stability, showed an increasing trend over the period 1975-2011 (APC = 3.4, 95% CI: 2.8 to 4.0, p < 0.001), which was reversed in the more recent decade 2011-2021 (APC = -6.1, 95% CI: -9.6 to -2.5, p = 0.001), with PAR (%) decreasing from a historical high of 19.3% in 2012 to 8.6% in 2021.

Conclusion

The high incidence of multiple births has a considerable impact on stillbirth rates in the Greek population. The recent downward trends of SBR and PAR (%) of multiple gestations are encouraging, however more measures and targeted interventions are needed to improve perinatal outcomes in multifetal gestation.

## Introduction

Stillbirth is a major and tragic complication of pregnancy, with devastating consequences for the families and the care providers. It is estimated that more than 2,000,000 fetal deaths occur annually worldwide, the majority of them in low- and middle-income countries, although stillbirth is still a major public health problem in developed countries as well [1-4].

In the epidemiological spectrum of fetal mortality, multiple gestation is a well-established causative factor. Clinical data suggest that twin pregnancies are associated with an enhanced risk of stillbirth, which may be five times higher for dizygotic twins and more than ten times higher for monozygotic twins, compared with single pregnancies, and the risk appears to increase further for higher-order multiples [5,6].

Several factors contribute to this increased risk. Multiple pregnancies are associated with higher risks of fetal growth restriction and congenital anomalies that increase the likelihood of fetal mortality, as well as placental complications such as placental abruption or twin-to-twin transfusion syndrome, potentially leading to fetal death [7-11]. Early detection of any potential complications and appropriate medical interventions are needed to reduce the risk of fetal mortality in these pregnancies [12,13].

Recently, an analysis of epidemiological data presented the notoriously high incidence of multiple pregnancies in Greece, which have reached epidemic proportions [14]. Thus, we sought to investigate the epidemiological burden of stillbirths conferred by multiple pregnancies in the Greek population, as well as the longitudinal trends.

## Materials and methods

Official national data regarding live births and stillbirths in Greece by multiplicity, based on the birth certificates registered in the country, were retrieved from the Hellenic Statistical Authority, covering a 65-year period from 1957 to 2021, the first and the most recent years with fully available data, respectively.

For each year of the above period, the stillbirth rate (SBR) was calculated separately for multiple, twin, triplet higher-order, and single births, defined as the number of stillbirths per 1,000 live births and stillbirths (total births). Stillbirths for the years 1957-1979 were defined as those that occurred at 28 weeks of gestation or more, whereas stillbirths for the 1980-2021 period were defined as fetal deaths occurring at 20 gestational weeks or more with a minimum fetal weight of 350 gr, following the definition suggested by the United States Centers for Disease Control and Prevention and the American College of Obstetricians and Gynecologists [15].

Also, the proportion of stillbirths from multiple pregnancies, and separately from twin, triplet, and higher-order pregnancies was calculated. Furthermore, the relative stillbirth risk for each type of multiplicity compared with single births, and triplet and higher-order multiple births compared with twins, was calculated as well, as the quotient of the corresponding stillbirth rates. Finally, the population attributable risk (%) (PAR (%)) was calculated using the formula [16]:

$PAR (%) = \{p(RR-1)}{p(RR-1)+1}$

p: prevalence of multiple births, calculated as the ratio of the number of total births derived from multiple births (live births and stillbirths) to the number of total births (live births and stillbirths).

Time trends were assessed using Joinpoint regression software version 4.7.0.0 (Surveillance Research Program, National Cancer Institute, Bethesda, Maryland, United States of America) to detect time points at which trends changed. The annual percentage change (APC) for each period was calculated, and the average APC (AAPC) for the entire 1957-2021 period was computed as a weighted average of the APCs, with the weights equal to the length of the APC interval, with a 95% confidence interval (95% CI) and a two-tailed level of statistical significance p<0.05.

Ethics board approval or consent procedures were not needed, since this was an analysis of national-aggregate publicly available data.

## Results

In the period 1957-2021, a total of 7,982,846 births were registered in Greece, of which 7,910,174 were live births and 72,672 stillbirths, with a total SBR = 9.1 per 1,000 total births. Among these, there were 7,742,332 single births, of which 7,676,489 were live births and 65,843 were stillbirths, with a total SBR = 8.5 per 1,000 total single births. Also, a total of 240,514 multiple births were registered, of which 233,685 were live births and 6,829 were stillbirths, resulting in a total SBR = 28.4 per 1,000 total multiple births.

**Figure 1:**
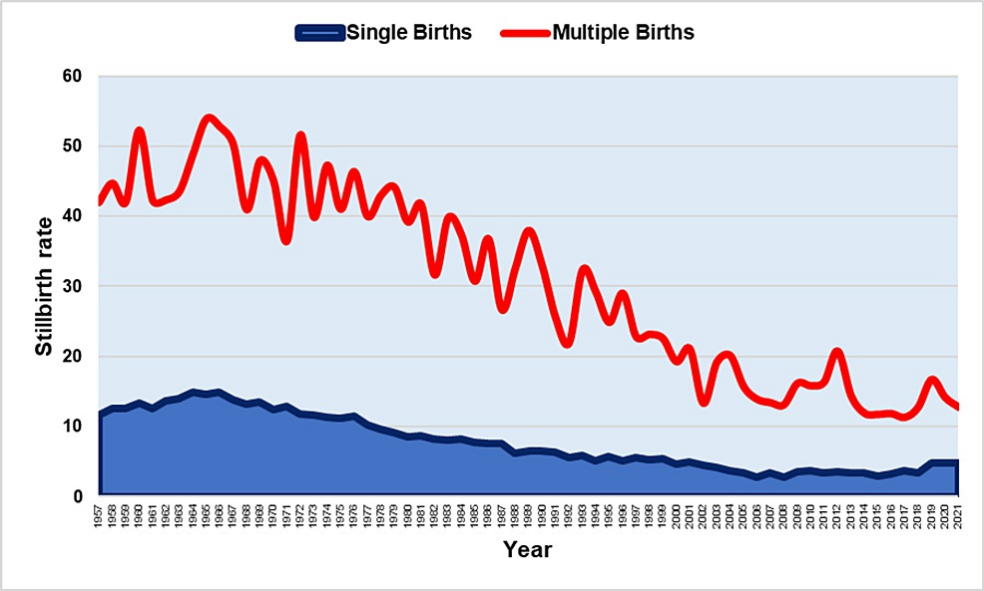
Stillbirth rates (per 1,000 births) in single and multiple births (1957-2021).

**Figure 2:**
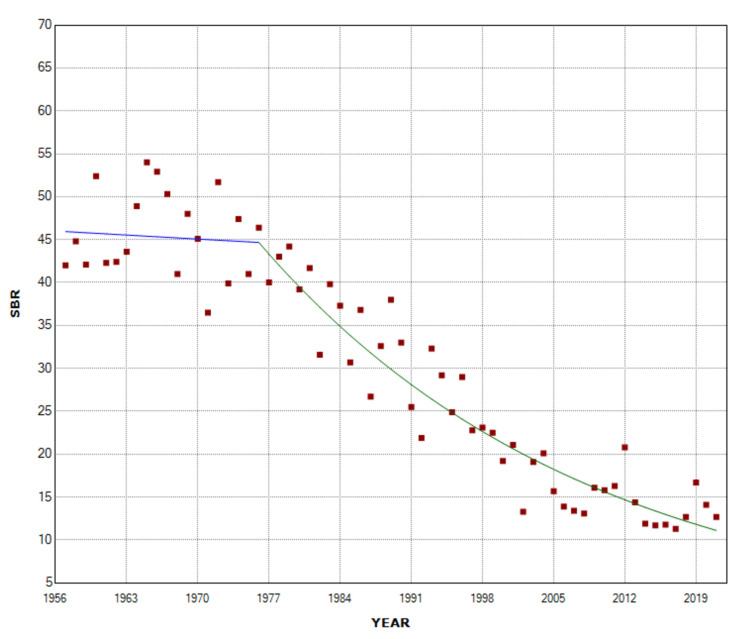
Time trends in stillbirth rate (SBR) in multiple births (1957-2021).

The SBR in multiple births remained unchanged from 1957 to 1976 (APC = -0.1, 95% CI: -1.4 to 1.1, p = 0.813) with a historically high SBR = 54.0 per 1,000 births in 1965 (28% higher than the SBR = 42.0 per 1,000 births in 1957), and showed a continuous downward trend from 1976 to 2021 (APC= -3.0, 95% CI: -3.4 to -2.7, p < 0.001), with a historic minimum of 11.3 per 1,000 births in 2017 (79% or 4.8-fold decrease compared with 1965) (Figures 1, 2, Table 1).

**Table 1:** Time trends in stillbirth rates in multiple, single, and twin births (1957-2021). APC: annual percentage change.

	Period	APC	95% CI	P-value
Multiple Births	1957-1976	-0.1	-1.4 to 1.1	0.813
	1976-2021	-3.0	-3.4 to -2.7	< 0.001
Single Births	1957-1965	2.9	0.8 to 5.2	0.009
	1965-2002	-3.2	-3.4 to -3.0	< 0.001
	2002-2005	-9.8	-25.8 to 9.7	0.295
	2005-2016	0.2	-1.3 to 1.7	0.819
	2016-2021	8.6	3.9 to 13.4	< 0.001
Twin Births	1957-1976	-0.1	-1.4 to 1.1	0.824
	1976-2021	-3.1	-3.4 to -2.8	< 0.001

**Figure 3:**
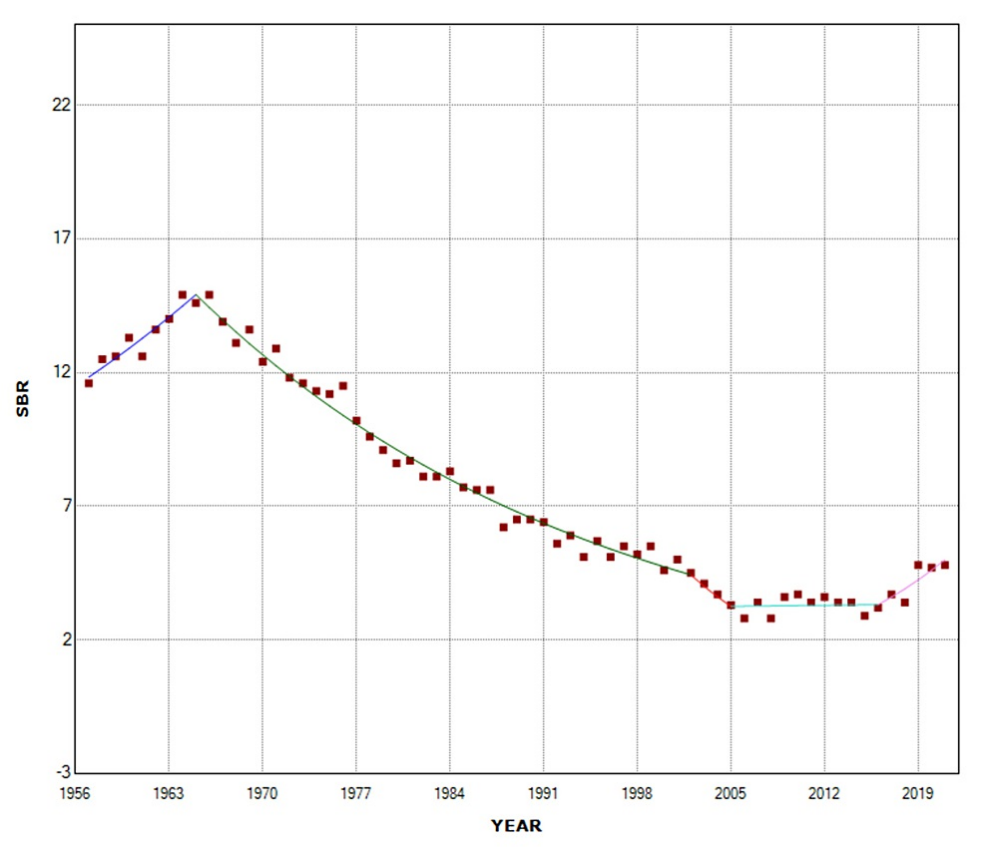
Time trends in stillbirth rate (SBR) in single births (1957-2021).

Among single births, the SBR showed an upward trend in the period 1957-1965 (APC = 2.9, 95% CI: 0.8 to 5.2, p = 0.009) and reached its maximum value in 1966 (14.9 per 1,000 births) 28% above the SBR = 11.6 per 1,000 births in 1957. After a long period of downward trend (1965-2002: APC = -3.2, 95% CI: -3.4 to -3.0, p < 0.001), followed by a stabilization in the period 2002-2016, with a historic minimum in 2008 (2.8 per 1,000 births, 81%, or 5.3-fold decrease compared with 1966), the SBR in singletons recorded a sharp increase in the recent period 2016-2021 (APC = 8.6, 95% CI: 3.9 to 13.4, p < 0.001). In 2021, the SBR in single births was 4.8 per 1,000 births, an increase of 71% from 2008 (Figures 1, 3, Table 1).

**Figure 4:**
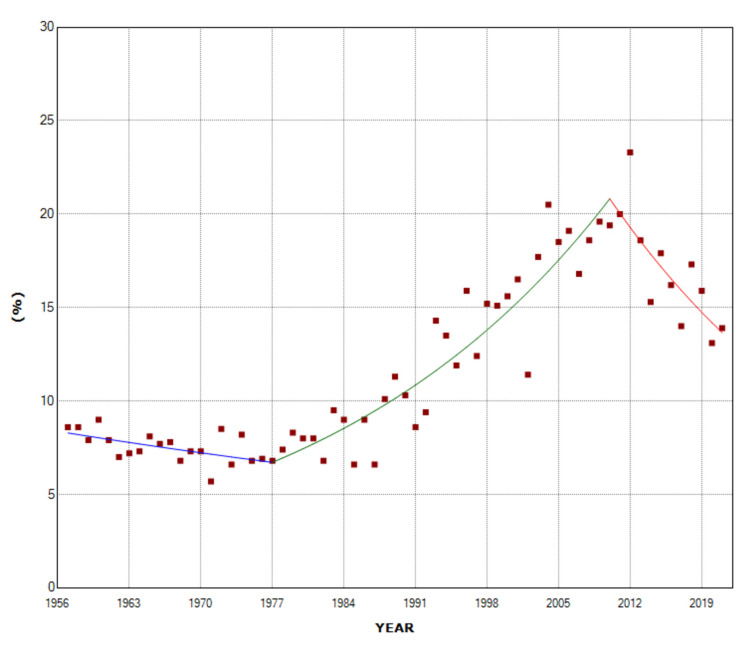
Time trends in the percentage of stillbirths from multiple births in the total stillbirths (1957-2021).

In the period 1957-2021, stillbirths from multiple pregnancies overall accounted for 9.4% of all stillbirths (6,289/72,672). The proportion of stillbirths from multiple births to total stillbirths declined slowly in the first two decades (1957-1977) with APC = -1.0 (95% CI: -2.0 to -0.0, p = 0.042) and reached a historic low in 1971 (5.7%, 110/1920), down 34% from 1957 (8.6%). Over the following decades to 2010, this proportion increased with APC = 3.5 (95% CI: 3.0 to 4.0, p < 0.001) to a historic maximum of 23.3% in 2012 (104/446) (4.1-fold higher than in 1971), but in the most recent decade, it steadily declined (2010-2021: APC = -3.8, 95% CI: -6.1 to -1.3, p = 0.003) to 13.9% in 2021 (63/453), a 40% decrease compared with 2012 (Figure 4, Table 2).

**Table 2:** Time trends in the percentage of stillbirths from multiple births in the total stillbirths (1957-2021). APC: annual percentage change.

Period	APC	95% CI	P-value
1957-1977	-1.0	-2.0 to -0.0	0.042
1977-2010	3.5	3.0 to 4.0	< 0.001
2010-2021	-3.8	-6.1 to -1.3	0.003

**Figure 5:**
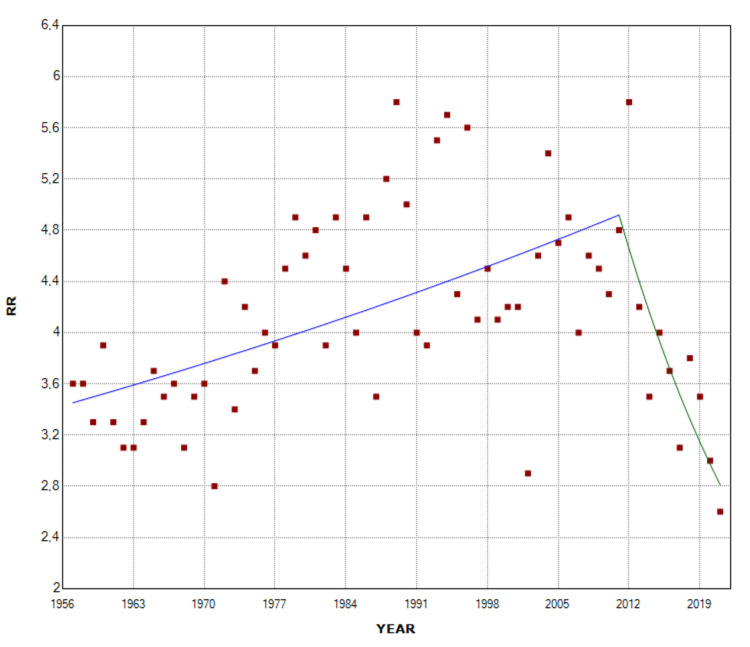
Relative risk (RR) for stillbirth of multiple compared with single births (1957-2021).

The overall incidence of intrauterine fetal demise was 3.34 times higher in multiple pregnancies compared with single pregnancies during 1957-2021 (RR = 3.34). The RR remained unchanged from 1957 to 2011, whereas in the last decade 2011-2021, it showed a statistically significant downward trend (APC = -5.4, 95% CI: -8.4 to -2.4, p < 0.001), after an all-time high of 5.8 in 2012. In 2021, the RR for stillbirth in multiple compared with singleton births reached a historic low of 2.6, down 55% from 2012 (Figure 5, Table 3).

**Table 3:** Time trends in the relative risk for stillbirth in multiple and twin births, compared with single births (1957-2021). APC: annual percentage change.

	Period	APC	95% CI	P-value
Multiple Births	1957-2011	0.7	0.4 to 0.9	< 0.001
	2011-2021	-5.4	-8.4 to -2.4	< 0.001
Twin Births	1957-1988	1.2	0.7 to 1.8	< 0.001
	1988-2012	-0.3	-1.1 to 0.5	0.459
	2012-2021	-4.8	-8.0 to -1.4	0.006

**Figure 6:**
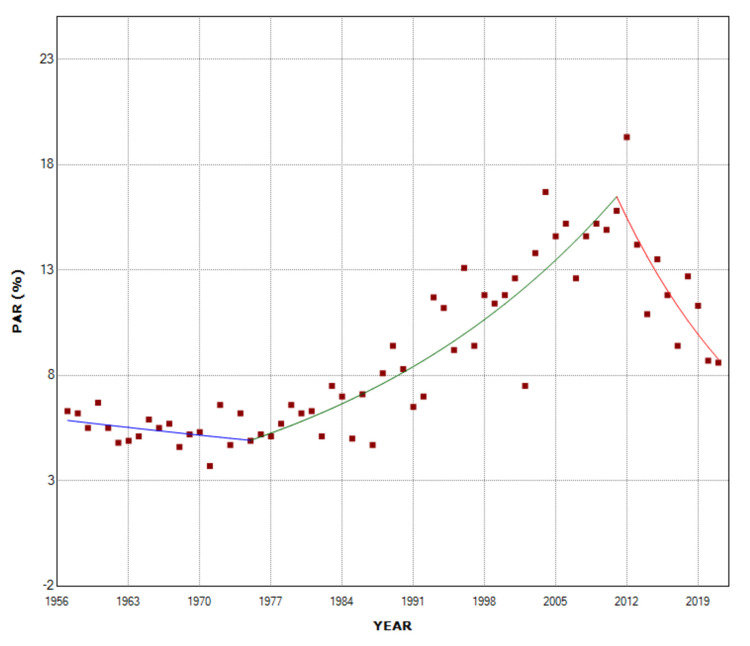
Time trends in the population attributable risk (%) (PAR (%)) for stillbirth of multiple births (1957-2021).

The overall PAR (%) of multiple births in stillbirths was 6.6% for the entire period 1957-2021. The PAR (%) did not change statistically significantly over the period 1957-1975 and reached a historic minimum of 3.7% in 1971. It then showed an upward trend in the period 1975-2011 (APC = 3.4, 95% CI: 2.8 to 4.0, p < 0.001) with the historically highest value of 19.3% in 2012 (5.2-fold higher than 1971), while the trend was downward in the most recent decade 2011-2021 (APC = -6.1, 95% CI: -9.6 to -2.5, p = 0.001). In 2021, the PAR (%) of multiple pregnancies in stillbirths was 8.6%, 55% down from 2012 (Figure 6, Table 4).

**Table 4:** Time trends in the population attributable risk (%) for stillbirth of multiple births (1957-2021). APC: annual percentage change.

Period	APC	95% CI	P-value
1957-1975	-1.0	-2.5 to 0.6	0.210
1975-2011	3.4	2.8 to 4.0	< 0.001
2011-2021	-6.1	-9.6 to -2.5	0.001

**Figure 7:**
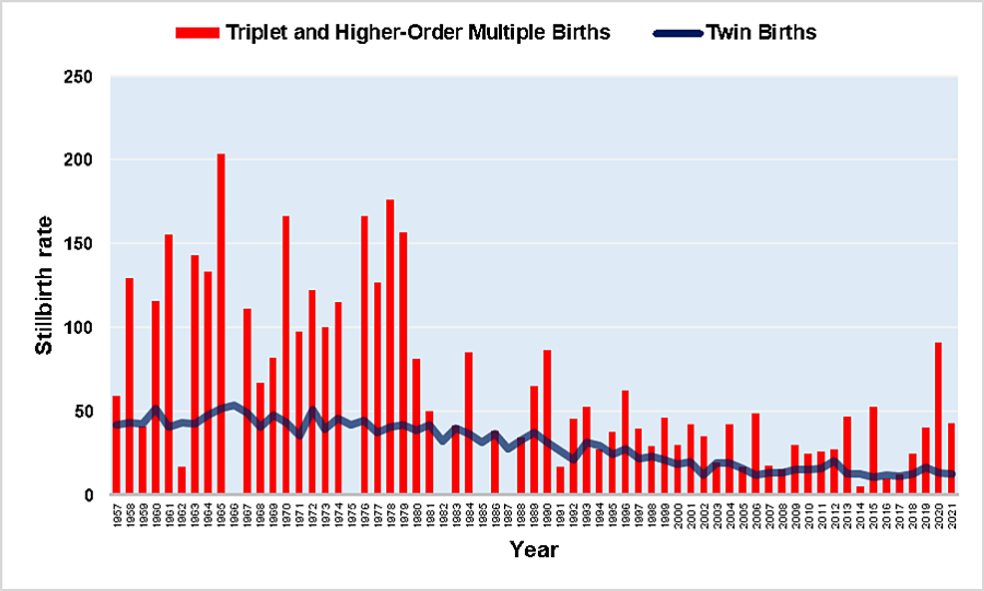
Stillbirth rates (per 1,000 births) in twin births, and triplet and higher-order multiple births (1957-2021).

**Figure 8:**
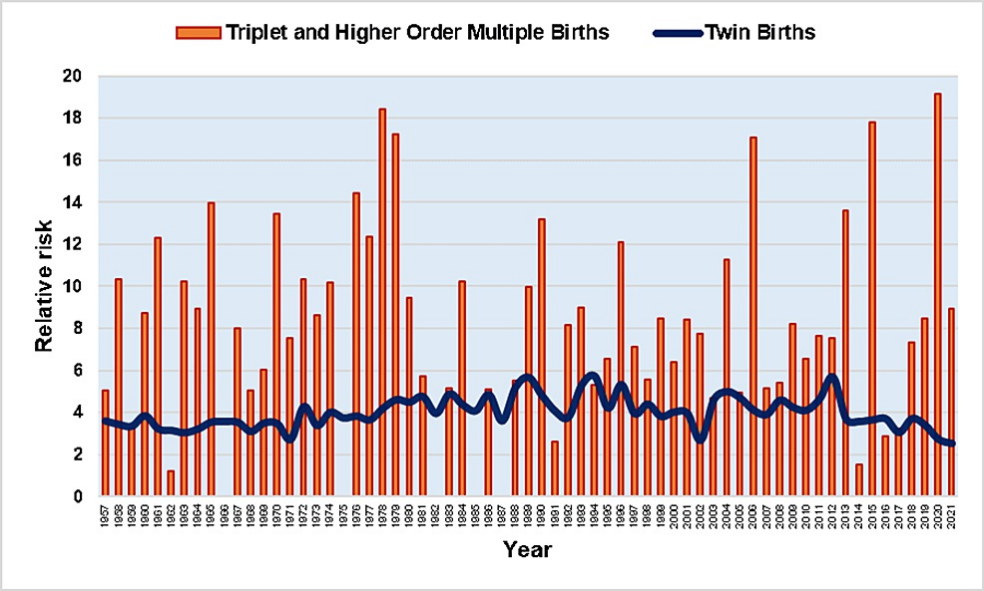
Relative risk of stillbirth in twin and triplet and higher-order multiple births compared with singletons (1957-2021).

**Figure 9:**
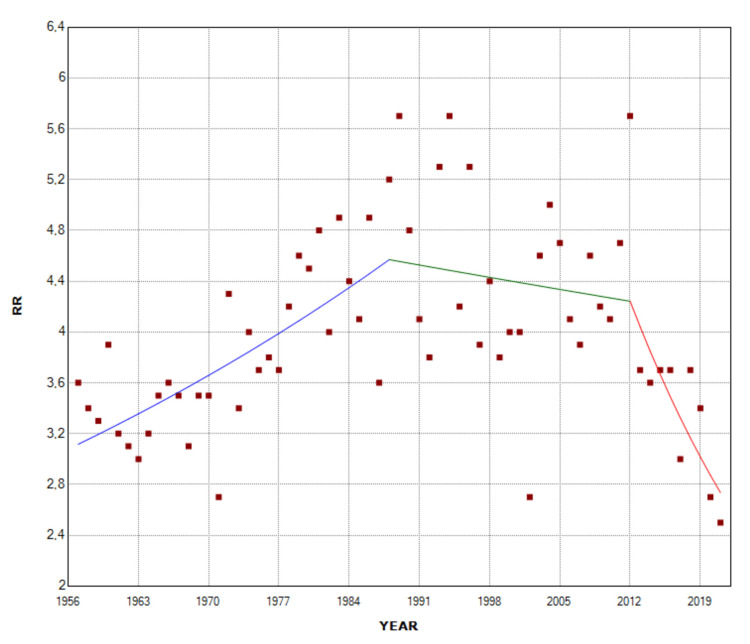
Trends in relative risk (RR) for stillbirth of twin compared with single births (1957-2021).

In twin births, the overall SBR was 27.8 per 1,000 births, with no statistically significant trend during 1957-1976 (APC = -0.1, 95% CI: -1.4 to 1.1, p = 0.824, and a clear downward trend from 1976 to 2021 (APC = -3.1, 95% CI: -3.4 to -2.8, p < 0.001). The RR of twins for stillbirth, compared with singletons, was 3.27 overall, ranging from RR = 5.7 in 2012 to RR = 2.5 in 2021, with an upward trend in the period 1957-1988 (APC = 1.2, 95% CI: 0.7 to 1.8, p < 0.001), a stabilization during 1988-2012, and a decreasing trend in the period 2012-2021 (APC = -4.8, 95% CI: -8.0 to -1.4, p = 0.006) (Figures 7-9, Tables 1, 3).

**Figure 10:**
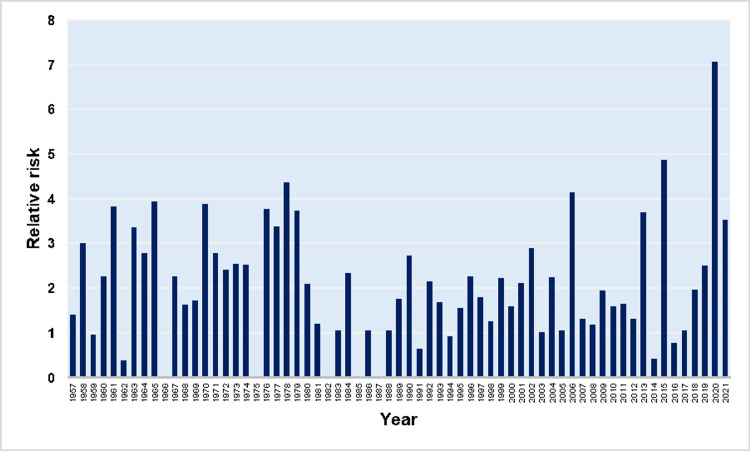
Relative risk for stillbirth of triplet and higher-order multiple births in comparison with twin births (1957-2021).

**Figure 11:**
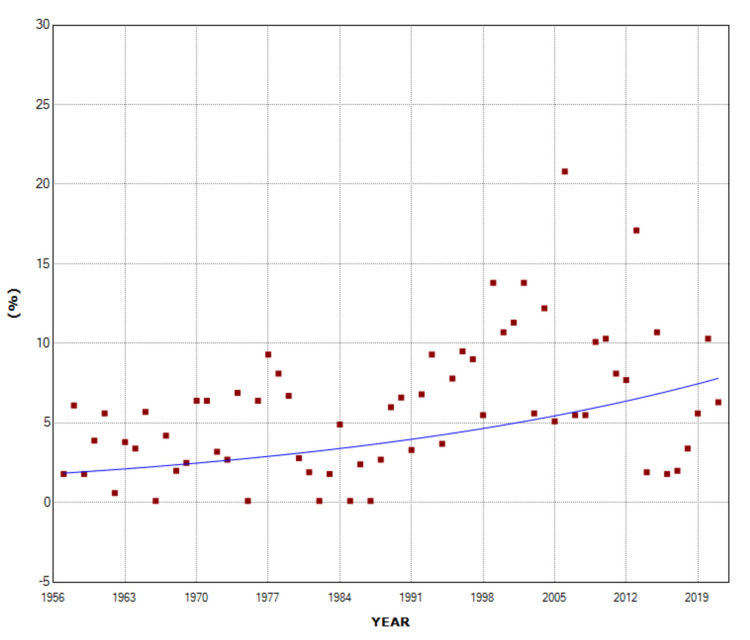
Time trend of the percentage of stillbirths derived from triplet and higher-order births to the total stillbirths from multiple births (1957-2021).

In triplet and higher-order multiple births, the overall SBR was 45.6 per 1,000 births, with substantial fluctuations from 0 (in the years 1966, 1975, 1982, 1985, and 1987) to an all-time high of 203.7 per 1,000 births in 1965, and no statistically significant trend over the period 1957-2021 (AAPC = -1.3, 95% CI: -3.7 to 1.1). The overall RR for stillbirth of triplet and higher-order multiple births, as compared with the singletons was 5.37, ranging from 0 to the historic high of 19.2 in 2020, without overall significant trend (AAPC = 0.8, 95% CI: -0.9 to 2.5). Comparing the incidence of fetal death between the two groups of multiple pregnancies, the risk was overall 64% higher for triplet and higher-order multiple pregnancies than twins. However, the SBR in triplet and higher-order multiple births was lower than in twins in 11 years (1959, 1962, 1966, 1975, 1982, 1985, 1987, 1991, 1994, 2014, and 2016). The RR for stillbirth for the triplet and higher-order multiple births, in comparison with the twin births, ranged from 0 to an all-time high of 7.1 in 2020, with no overall statistically significant trend (AAPC = 0.3, 95% CI: -0.9 to 1.6). Finally, the percentage of stillbirths derived from triplet and higher-order births to the total stillbirths from multiple births was 5.4% for the entire period 1957-2021. This proportion reached its maximum of 20.8% in 2006 and exhibited an overall rising trend (AAPC = 2.3, 95% CI: 0.7 to 3.9) (Figures 7, 8, 10, 11).

## Discussion

The present study highlights the high risk of fetal mortality in multiple pregnancies and quantitatively documents this association over time in the Greek population. Fetal mortality in singleton and multiple pregnancies and their evolutionary trends over a 65-year period, from 1957 to 2021, were investigated separately to determine their distinct contribution to the overall SBR in Greece. Our results showed that overall, over the entire period under consideration, SBR was 3.34 times higher in multiple pregnancies compared with single births (28.4 vs 8.5 per 1,000 total births, respectively).

Fetal mortality in multiple pregnancies has declined steadily over the past four decades with a historic low of 11.3 per 1,000 births in 2017. The overall SBR in the Greek population started to increase after 2008, with the onset of the economic crisis in the country [15,17]. Our analysis indicated that the deterioration of fetal mortality in Greece after the financial crisis is essentially the result of an increase in fetal mortality in singleton pregnancies.

Our results showed that multiple pregnancies contribute significantly to the total number of stillbirths in Greece. During the 65 years examined, multiple pregnancies overall accounted for 9.4% of fetal deaths, with a wide variation, from 5.7% in 1971 to a staggering 23.3% in 2012. This figure undoubtedly followed the parallel dramatic increase in the multiple birth rate in Greece [14]. However, in the last decade, while the epidemic of multiple pregnancies has continued to gigantically grow as a major public health problem in the country, specifically of twin births, fewer stillbirths came from multiple gestations, 13.9% in 2021, down 40% compared with 2012.

The comparison of SBR between single and multiple pregnancies allowed the RR of fetal death per year to be calculated, as well as the mapping of longitudinal trends. While the RR of stillbirth in multiple compared with single births has been stable for more than five decades, it has decreased drastically in the last decade, from 5.8 in 2012 to 2.6 in 2021, apparently reflecting more effective antenatal care of multiple pregnancies and control of key risk factors in the Greek population [9,18]. Notably, similar recent improvements in multiple stillbirth rates have been also reported in other countries [19].

The most remarkable finding of the present study is the calculation of the population risk of fetal death attributable to multiple births in the Greek population. The PAR (%) is an extremely important indicator from a public health perspective and expresses the proportion of the incidence of the disorder that is due to exposure to a specific risk factor. The PAR (%) is the proportion of the incidence of fetal deaths that would be eliminated if the risk in multiple gestations were equal to that in singletons, i.e., it measures the expected benefit of eradicating the risk factor in the population. According to our analysis, the overall PAR (%) was 6.6% for the entire period 1957-2021, with large variation, from a minimum of 3.7% in 1971 to a hefty 19.3% in 2012. The PAR (%) showed a remarkable downward trend over the last decade, reaching 8.6% in 2021. The multiple birth PAR (%) is determined by two parameters: the prevalence of multiple births and the RR of fetal death in multiple pregnancies compared to singletons. The steep decline in multiple birth PAR (%) in the last decade in Greece is evidently attributed entirely to the decline in RR since the multiple birth rate continued to rise [14].

Despite the large decrease over the last decade, the (%) population stillbirth risk attributable to multiple pregnancies in the country remains sizeable. One study analyzed data on stillbirth (≥ 28 gestational weeks) from 29 European countries in the Euro-Peristat project (not including Greece) for the year 2010, and the estimated pooled RR for fetal death among multiple pregnancies, as compared with singletons, was 2.4, ranging from 0.8 in Denmark to 5.7 in Wallonia (Belgium), whereas the estimated total PAR (%) for multiple fetal mortality was 4.4% [20]. The impact of multiple pregnancies on stillbirth rates in Greece has been insufficiently evaluated.

In a study examining the determinants of stillbirth in Greece in the period 1989-1991 after analysis of national data of all stillbirths in the country at gestational age greater than 28 weeks compared with a random sample of 30,705 live births from the same period, a crude odds ratio 4.8 was reported [21], similar to an odds ratio of 5.1 that could be calculated with the data of the present analysis for this period.

In contrast, in a more recent study of national data examining stillbirth at ≥26 weeks of gestation in Greece in 2004-2015, under a multivariable model, the authors reported a high RR of stillbirth in multiple births compared with singletons, specifically RR = 20.1 for the Greek women and a lower RR = 10.2 for the non-Greek women [22]. Our analysis of all stillbirths occurring after 20 weeks of gestation for the 2004-2015 period resulted in an overall RR = 4.6.

Finally, the SBR was examined separately in twins, triplets, and higher-order pregnancies. Overall, twin pregnancies had a 3.27 times higher risk of stillbirth, compared with singletons, with decreasing trends over the last decade, while for triplet and higher-order births, the corresponding overall RR was 5.37. In the last decade stillbirths from triplet and higher-order pregnancies have constituted a decreasing proportion of total multiple stillbirths due to the relative decrease in the prevalence of these pregnancies [14].

The advantage of our study is the analysis of national data for the whole population, which are collected reliably and systematically by the Hellenic Statistical Authority based on birth certificates for which there is a legal obligation to complete. A limitation is the univariate analysis, because part of the association between multiple births and stillbirth may be mediated by other confounding factors, such as maternal age. Nevertheless, the reported RRs and PARs (%), as well as the secular trends retain their relevance for the descriptive epidemiology of fetal mortality in Greece.

## Conclusions

Multiple pregnancies have a considerable impact on stillbirth rates in the Greek population. Our analysis indicated an increasing impact of multiple pregnancies on fetal mortality at the population level over a long period in parallel with the rise in multiple births in the country, which was reversed by an encouraging recent steep downward trend of PAR (%) for fetal deaths of multiple gestations. However, more progress is needed, toward the adoption of recommended practices to reduce the incidence of multifetal pregnancies following assisted reproductive technologies, and integrated antenatal care with tailored interventions to address preventive risks for fetal deaths in multiple pregnancies in Greece.
